# The pharmacological treatment of acute vestibular syndrome

**DOI:** 10.3389/fneur.2022.999112

**Published:** 2022-09-09

**Authors:** Pasquale Viola, Federico Maria Gioacchini, Alessia Astorina, Davide Pisani, Alfonso Scarpa, Gianmarco Marcianò, Alessandro Casarella, Emanuele Basile, Vincenzo Rania, Massimo Re, Giuseppe Chiarella

**Affiliations:** ^1^Unit of Audiology, Department of Experimental and Clinical Medicine, Regional Centre of Cochlear Implants and ENT Diseases, Magna Graecia University, Catanzaro, Italy; ^2^ENT Unit, Department of Clinical and Molecular Sciences, Polytechnic University of Marche, Ancona, Italy; ^3^Department of Medicine and Surgery, University of Salerno, Salerno, Italy; ^4^Department of Health Science, School of Medicine, University “Magna Graecia” of Catanzaro, Catanzaro, Italy

**Keywords:** acute vestibular syndrome, vestibular neuritis, posterior circulation stroke, vertigo, pharmacologic treatment

## Abstract

Acute vestibular syndrome (AVS) represents a clinical picture that involves urgent management due to the important procession of symptoms accompanying the event, which can be positively or negatively influenced by therapeutic choices and intervention timing. This forces a differential diagnosis and therapeutic choices to be made in conditions that are not always favorable and often not in the specialist field. In this work, we will examine in detail the pharmacological therapeutic possibilities, correlating them to the differential and, as far as possible, to the etiological diagnosis. In particular, the pharmacological possibilities for the two main conditions we can face will be investigated, namely, vestibular neuritis and posterior circulation stroke.

## Introduction

Vertigo and/or dizziness are frequently reported in patients admitted to the emergency department (ED), accounting for nearly 4% of admissions (1). There is a wide spectrum of causes including cardiovascular, neurological, vestibular, and systemic disorders (2). It has been observed that 10–20% of the patients admitted to ED due to persistent vertigo and dizziness have an acute vestibular syndrome (AVS) (3). AVS is defined as the sudden onset of acute, “continuous” vertigo (lasting longer than 24 h), associated with nausea, vomiting and head motion intolerance, gait instability, and nystagmus (ny). It results from a unilateral vestibular lesion that causes a sudden asymmetry of the neuronal nuclei firing rate and is largely associated with severe anxiety and vasovagal responses (2). The main causes of AVS are vestibular neuritis (VN), which nearly counts for 70% of cases, and posterior circulation stroke (PCS), accounting for 25% of diagnoses (1, 4). Discrimination between these pathologies is necessary for correct patient management, but despite a large investment of resources, PCS still too often escapes diagnosis and is sometimes missed (5). Approximately 10–20% of spontaneous AVS are due to stroke in the brainstem or cerebellum, nevertheless fewer than 20% present with focal neurological signs (6, 7). VN is the third most common, peripheral vestibular disorder, after benign positional paroxysmal vertigo (BPPV) and Meniere's disease (MD). The leading hypothesis involves reactivation of a latent neurotropic virus, for example, herpes simplex virus (HSV) types 1 and 2, and herpes zoster virus (HZV). VN has recently been related to severe acute respiratory syndrome coronavirus-2 (SARS-CoV-2) infection, but this association is still uncertain and definitive evidence is lacking (8–10). Other supposed etiologies include vascular, immunologic, and inflammatory (11, 12). Stroke affects the brain circle, resulting in the death of neurons, according to oxygen and nutrients deprivation (13). Ischemic stroke is more likely to determine AVS, compared to hemorrhagic stroke. The stroke involves more frequently posterior inferior cerebellar artery (PICA) than anterior inferior cerebellar artery (AICA) (14). Some patients with AVS can show focal lesions in the nodulus of the cerebellum, in the cerebellar peduncles, in the dorsolateral pons, in the lateral medulla, in the root of the eighth cranial nerve at the pontomedullary junction, or in the vestibular nuclei (15–19). Other possible causes of AVS are MD, multiple sclerosis, thiamine deficiency, BPPV, and vestibular migraine. Multiple sclerosis is an uncommon and infrequent cause of acute vertigo (4%). Demyelinating plaques can be located in and around the eighth nerve fascicle or vestibular nuclei but also in the brainstem and cerebellar peduncles. Considering that demyelinating lesions during an acute attack may not be evident on MRI, clinical examination in AVS is essential. In these patients, more evident oculomotor signs are often present (limitation of ocular motility or vertical nystagmus) (20).

Thiamine deficiency underlying Wernicke encephalopathy (WE) should be considered in patients with nutritional deprivation and unexplained acute or subacute vestibular symptoms, even absent encephalopathy. The complete WE triad includes ophthalmoplegia, ataxia, and encephalopathy. In about 90% of cases, there is altered mental status and nystagmus with central features. Nystagmus was the only ocular feature in 65% of cases. The most common human vestibular finding in WE is bilateral vestibular hypofunction, occurring in about 90% of cases. In the pre-encephalopathy phase, thiamine-deficient patients presenting with predominantly vestibular symptoms and signs can mimic vestibular neuritis or stroke with acute, persistent, vertigo, severe vomiting, and gait ataxia for 48 h. MRI of the brain in the pre-encephalopathy phase may be normal. Subsequently, it can present alterations consistent with WE such as areas of increased fluid-attenuated inversion recovery/T2 signal in the midline thalami, upper midbrain, and pons. Vestibulopathy in WE disease is due to direct bilateral damage of brainstem most likely in the dorsal medulla and pons in the region of the medial vestibular nuclei and nucleus prepositus hypoglossi in the medulla. The therapy involves the administration of vitamin B1 which in most cases determines an improvement in the patient's condition (21, 22). However, these clinical entities are less frequent than VN and stroke in AVS-like presentation and should be held in account when other causes are not identified (5).

## Differential diagnosis

Correctly distinguishing between VN and PCS is essential although it is not always easy, due to the possible similar clinical presentation. Hemiparesis, headache, diplopia, dysarthria, and ataxia are neurological deficits that may be signs of PCS; however, they are not present in all patients (2, 23). In stroke suspicion, anamnesis has a key role. Smoke, diabetes mellitus, hypertension, hypercholesterolemia, diet, physical activity, and cardiovascular disease are important clues (24). VN ny is generally horizontal, maintains the same direction after changing gaze side, and is attenuated by visual fixation. Conversely, ny of central origin is multidirectional, and its intensity is less affected by visual fixation. Although horizontal mixed torsional ny is possible in both central and peripheral pathologies, vertical or pure torsional ny is suggestive of a central origin. Negative clinical head impulse test (HIT), direction changing ny, and skew deviation (HINTS) are suggestive of central origin. HINTS test is highly sensitive and specific in detecting vestibular strokes, and it outperforms acute magnetic resonance imaging (MRI) within 48 h from symptoms onset (6, 25). MRI diagnosis of smaller strokes (<1 cm) indeed can fail up to 50% of the cases within the first 24 h (26). ABCD2 score is also useful to quantify the risk of stroke, evaluating age, blood pressure, clinical features, duration, and diabetes in subjects (27). In the acute setting, computed tomography (CT) is more useful to evaluate a patient with suspected stroke, because it is quicker and easily available. MRI is more sensible in detecting early signs of ischemia. Brain imaging is essential in patients with suspected PCS and who may need thrombolysis or thrombectomy. These choices should be made based on risk factors, signs, and symptoms (2, 23). MRI has been shown to be effective in detecting signs of recent PCS onset, and it, therefore, allows patients to be referred for thrombolysis within the time interval suggested by the guidelines (28, 29). The symptoms and signs in patients with an AVS associated with a stroke commonly evolve over hours and often require frequent monitoring. In patients with the AVS, the finding of ocular lateral deviation (OLD), although infrequent (8.4%), usually reflects lateral medullary syndrome (LMS), particularly when associated with hypometric corrective saccades on opening the eyes. OLD is a conjugate, ipsilesional, horizontal ocular deviation associated with brief (3–5 s) closing of the eyes that is highly specific for a central disturbance. OLD is easily tested at the bedside and can be a quick confirmatory sign when patients have a HINTS pattern of eye movements suggesting a central cause, particularly when initial imaging is negative. To maintain specificity, a complete horizontal deviation must be present after a brief period (3–5 s) of simply closing the eyes. Clinicians should look for OLD with brief, gentle eyelid closure and for a series of hypometric, corrective saccades back to straight ahead on opening the eyes. Both findings point to a central lesion that usually is in the lateral medulla on the same side as the OLD (30).

Also, the “STANDING” appears to show high sensitivity and specificity to detect central vestibulopathy, with good reliability in the emergency setting, and seems to be associated with a reduction of neuroimaging burden and hospital admission rates. STANDING is an acronym for the four-step clinical algorithm based on ny observation, and diagnostic maneuvers (Dix-Hallpike and Pagnini-McClure positionings) include the discrimination between SponTAneous and positional nystagmus, the evaluation of the Nystagmus Direction, the head Impulse test, and the evaluation of equilibrium (staNdinG) (31).

Larger cerebellar strokes (usually PICA, or less commonly SCA territory) with only vestibular and ocular motor signs need close monitoring in the intensive care unit for the development of malignant ischemic edema, which may be delayed at times for several days. Treatment may require hypertonic saline or different surgical interventions (external ventricular drain) or resection of necrotic tissue with good post outcome. Videonystagmography, electronystagmography, and/or vestibular evoked myogenic potentials (VEMPs) are useful to better qualify and quantify vestibular deficit (26).

## Pharmacological treatment

### Vestibular neuritis

The VN pharmacological management is aimed to reduce symptoms and inflammation in the acute phase. In fact, most people undergo complete resolution, but imbalance may last for weeks (11, 32, 33). Vestibular suppressants and antiemetics are useful for short intervals of time. If their administration is prolonged, they may obstacle VC (11, 26). In the acute setting, intravenous dimenhydrinate showed a major efficacy compared to intravenous lorazepam, in a randomized clinical trial by Marill and colleagues, in 74 patients (34). Antihistamines, benzodiazepines, anticholinergics, and dopamine receptor antagonists are possible therapeutic options in the first 2–3 days (33, 35). Corticosteroids use in VN is a controversial topic (36). Fishman et al. showed the absence of a long-term effect on symptoms. These compounds had a significant effect only on 1-month-performed caloric test (37). Other authors confirmed these results (38, 39). However, their use is a matter of fact and may provide symptoms relief in patients in the first 72 h (11, 26). Goudakos et al. experimental results sustain an earlier corticosteroids efficacy. However, the study was single blinded and had limitations (39). Vestibular rehabilitation has an important role in patient with long-term symptoms and seems to be comparable to corticosteroids in the main early outcomes (11, 33, 39). Nutraceuticals, including *Ginkgo biloba, Salvia officinalis, Melissa officinalis*, and *Zingiber officinalis*, may improve patient's conditions with a low amount of side effects (12). We previously treated these drugs' mechanism of actions, interactions, and side effects in a narrative review (35). Further details are summarized in Tables 1–3.

**Table 1:** Mechanism of action and dosage of vestibular neuritis drugs.

	**Mechanism(s) of action**	**Dosage suggested**	**Route of administration**
Betahistine	Strong antagonist of histamine H3 receptors and a weak agonist of H1 receptors (40, 41)	24/48 mg daily (40)	OS (40)
Benzodiazepines
Diazepam	Allosteric modulation of GABA_A_ receptor (42, 43)	4–60 mg/daily (OS) 10–60 mg/daily (IV, IM) (44)	OS, IV, IM, rectal (43)
Lorazepam	Allosteric modulation of GABA_A_ receptor (42, 43)	2–10 mg/daily (45, 46)	OS, IM, IV (43)
Anticholinergics
Atropine	Non-selective muscarinic blocker (47)	0, 3–4 mg (depending on clinical indication) (47, 48)	IV, IM, SC (47, 48)
Glycopyrrolate	Non-selective muscarinic blocker (49)	2 mg in clinical trial (50), but may vary depending on clinical indication 1–8 mg (OS) (51) Various (parenteral) (49, 52)	IV, OS, IM (49, 51, 53)
Scopolamine	Non-selective muscarinic blocker (54)	0, 25–1 mg daily (IM,IV) (54) 0, 5 mg (TD) in clinical trial (55)	IM, IV, TD (54, 55)
Antihistamines
Dimenhydrinate + cinnarizine	D: antagonist of H1 receptor (56) C: It blocks voltage-gated calcium channels, preventing calcium translocations across the vestibular air cells and, thus, regulating hair cell afferent vestibular transmission, anti-vasoconstrictor activity, reduction in the blood viscosity of the inner ear's circulatory system (56)	Dimenhydrinate: 25–200 mg (OS) (57) Basis of 50 mg (IV-IM), but may vary (58) Cinnarizine: 15–225 mg (OS) (59, 60) Recommended clinical dose (vestibular disorders): varies between 25 mg thrice-daily and 75 mg once-daily, up to a maximum of 225 mg (60) Dimenhydrinate/cinnarizine (co-formulation): 20/40 mg thrice a day (61)	IV, IM, OS (d) (57, 58) OS (c) (59) OS (co-formulation) (62)
Diphenhydramine	Antagonist of H1 receptor (62, 63)	25 mg-50 mg (62, 64, 65)	OS (62, 64)
Meclizine	Antagonist of H1 receptor (66)	12, 5–25 mg (67, 68)	OS (67)
Promethazine	Antagonist of H1 receptor (68, 69)	25–100 mg (OS) 25–50 mg; max:100 mg (IM,IV) (69) May vary (70)	OS, IM, IV (69)
Other antiemetics			
Metoclopramide	It acts on 5HT4 (agonist), 5HT3 (antagonist) and dopamine D_2_ (antagonist) receptors (43, 70, 71)	10–30 mg or max 0, 5 mg/kg (IV-IM-OS) (71, 72)	OS, IM, IV, rectal (71, 72)
Ondansetron	5HT3 antagonist (73, 74)	4–8 mg capsules (OS), multiple administration also 8 mg (IV-IM) 16 mg (rectal) (74)	OS, IM IV, rectal (74)

GABA, gamma aminobutyric acid; IM, intramuscular; IV, intravenous; OS, oral; SC, subcutaneous; TD, transdermal.

**Table 2:** Pharmacokinetics of drugs used in peripheral vestibular vertigo (part I).

	**Oral Bioavailability**	**Time to Peak Concentration**	**Serum Half-life (t1/2)**	**Protein Binding**	**Transporter proteins**	**Metabolism**	**Metabolites**
BHS	NA	1 h	3.5 h	5%	-	Monoamine oxidases (MAO) A/B	2-pyridylacetic acid (2-PAA)
BDZ							
CLZ	90%	1.2 h	23 ± 5 h	82–86%	-	Liver (glucuronidation), CYP3A4	7-aminoclonazepam and 7-acetamido-clonazepam
DZP	90–100%	0.5–1.5 h	24–48 h	96–98%	-	Liver (glucuronidatio), CYP3A4, CYP2C19	Desmethyldiazepam, oxazepam, temazepam
LOR	90%	2–3 h	12–16h	85–90%	-	Liver (glucuronidatio)	3-O-phenolic glucuronide
ACDs							
ATP	-	10 min (IM)	4 h	-	-	50% liver 50% unmodified	NA
GLY	3% (children) NA, but higher (adults)	NA	0.83 ± 0.27 h (IV) 75 min (IM) 2.5–4 h (OS, solution)	-	-	NA	NA
SCO	NA	2 min (IM) 24h (TD)	8 h	-	-	Hepatic	NA
AHs (H1 antagonists)							
DIM + CNZ	43–72% (d)	1–4h (d); 2–4h (c)	6–7h (d); 4–5h (c)	80–85% (d)		Hepatic (d, *see the section below*). CYP2D6 and CYP2B6, but other CYP may be involved (c)	D: diphenhydramine, DMDP; C: conjugated with glucuronic acid
DPH	43–72%	1–4h	3–9.3 h	80–85%	-	Hepatic first-pass metabolism CYP2D6, and to a minor extent CYP1A2, CYP2C9 and CYP2C19	DMDP
MEC	NA	1.5–6h	5.21 ± 0.80 h	NA	-	Hepatic CYP2D6	Norchlorcyclizine (rats), 10 different metabolites in human urines. Human metabolites have not been identified, but meclizine undergoes aromatic hydroxylation or benzylic oxidation.
PMZ	25%	2–3h	4–6h (OS) 9–16 (IV) 6-13 (IM)	-	-	Hepatic first-pass metabolism	Promethazine sulfoxide (PMZSO), N-demethylpromethazine
Other antiemetics							
MCP	35–100%	0.5–2h (OS); 3 h (IM)	5–6h	13–40%	-	Hepatic: CYP2D6 isoform, and possibly CYP1A2 and CYP3A; conjugation.	Argikar et al. identified 10 metabolites of metoclopramide (M1-M10) in the urine after oral administration. Of those (M1, M2, M6, M7, and M8) were conjugated to either glucuronide or sulfate. Mono-de-ethyl-metoclopramide and N-4 sulfate conjugated are two important products.
OND	56% (OS); 60% (rectal)	1.5 h (OS); 6 h (rectal)	3–6 h	70–76%	P-gp substrate	Hepatic first-pass metabolism, CYP1A2, CYP2D6, CYP3A4	Hydroxylation on the indole ring followed by subsequent glucuronide or sulfate conjugation.

ACDs, anticholinergic drugs; AHs, antihistamines; ATP, atropine; BDZ, benzodiazepines; BHS, betahistine; CLZ, clonazepam; CNZ, cinnarizine; CYP, cytochromes P450; DIM, dimenhydrinate; DMDP, monodesmethyldiphenhydramine; DPH, diphenhydramine; DZP, diazepam; GLY, glycopyrrolate; IM, intramuscular; IV, intravenous; LOR, lorazepam; MAO, monoamine oxidase; MCP, metoclopramide; MEC, meclizine; NA, not available; OND, ondansetron; OS, oral; P-gp, p-glycoprotein; PMZ, promethazine; SCO, scopolamine; TD, transdermal.

**Table 3:** Pharmacokinetics of drugs used in peripheral vestibular vertigo (part II).

	**Enzymes Inductor/Inhibitor**	**Elimination**	**Dose Changes in Hepatic Disease**	**Dose Changes in Renal Disease**	**References**
BHS	-	85% urine Low levels in bile	No dosage adjustment seems to be needed	No dosage adjustment seems to be needed	(40, 75)
**BDZs**					
CLZ	-	50–70 % in urine 10–30 % feces	Protein binding may be changed by cirrhosis, increasing the free fraction. Caution needed. Contraindicated in severe hepatic impairment.	Caution needed	(43, 45, 76)
DZP	-	100% urine	Contraindicated in severe hepatic impairment. Caution needed in other mild and moderate hepatic impairment	Caution needed	(43–45, 77)
LOR	-	88 ± 4% urine 7± 2% feces.	Caution needed. Contraindicated in severe hepatic impairment	Caution needed in severe hepatic impairment	(45, 46, 78, 79)
ACDs					
ATP	-	50% liver 50% urine	Caution needed	Caution needed	(47, 48, 80)
GLY	-	Urine, only 5% bile	Further studies needed. Since kidney elimination has a major role, hepatic impairment seems not to be relevant, despite a certain negative effect of anticholinergic drugs on hepatic damage.	Dose reduction by 30% in patients with mild to moderate renal impairment. Contraindicated in severe renal impairment.	(49, 52, 81)
SCO	-	Urine	Caution needed for the risk of CNS reactions	Caution needed for the risk of CNS reactions	(54, 80, 82)
**AHs (H1-antagonists)**					
DIM + CNZ	Inhibition of CYP2D6 (d)	Mainly in urine (d)	Caution needed (d)	Caution needed (d)	(57, 61, 62, 83, 84)
		40–60% feces and minor quote in urines (c)	Coadministration contraindicated in patients with severe hepatic impairment	Coadministration contraindicated in patients with eGFR <25 ml/min	
(DPH	It inhibits CYP2D6	Mainly in urine	Caution needed	Caution needed	(62, 70, 85–87)
MEC	Meclizine seems to reduce the expression of CYP2B10, 3A11, 1A2 in experimental models	Urine, feces	Caution needed (need further evaluation)	Caution needed (need further evaluation)	(66, 88, 89)
PMZ	-	Urine	Caution needed (need further evaluation)	Caution needed (need further evaluation)	(69, 70, 90, 91)
**Other antiemetics**					
MCP	-	86% urine, minor quote in bile	Caution needed	Caution needed In patients with last stage renal impairment (eGFR ≤ 15 ml/min): dose reduction of 75%. Severe/moderate renal impairment (eGFR 15-60 ml/min): dose reduction of 50%.	(70–72, 92, 93)
OND	-	Majority hepatic, 5% urine	Caution needed, especially in severe hepatic impairment	Caution needed, although studies on moderate renal impairment did not show significant changes	(43, 73, 74, 94, 95)

ACDs, anticholinergic drugs; AHs, antihistamines; ALT, alanine aminotransferase; ATP, atropine; BDZ, benzodiazepines; BHS, betahistine; BUN, blood urea nitrogen; CLZ, clonazepam; CNS, central nervous system; CNZ, cinnarizine; CYP, cytochromes P450; DIM, dimenhydrinate; DPH, diphenhydramine; DZP, diazepam; eGFR, estimated glomerular filtration rate; GLY, glycopyrrolate; LOR, lorazepam; MCP, metoclopramide; MEC, meclizine; NIH, National Institutes of Health; NA, not available; OND, ondansetron; P-gp, P-glycoprotein; SCO, scopolamine; PMZ, promethazine.

### Posterior circulation stroke

#### Tissue plasminogen activators

Alteplase and tenecteplase are tissue plasminogen activators. Their main activity consists in the conversion of plasminogen to plasmin: this action is responsible for fibrinolysis (96, 97). Alteplase IV administration (in patients ≥ 18 years: 0.9 mg/kg, maximum dose 90 mg over 60 min; in the beginning, 10% of dose must be given as a bolus over 1 min) is recommended until 4.5 h after stroke onset. Treatment should be started as soon as possible. In severe stroke, after 3–4.5 h from symptoms onset, some categories of patients must be excluded (or have a lower evidence indication) for the high risk of hemorrhage: patients >80 years; combined history of diabetes–stroke; warfarin assumption, without considering international normalized ratio (INR); and very severe stroke with National Institutes of Health Stroke Scale (NIHSS) > 25 (29, 98). Alteplase is recommended for patients with mild stroke and mild disabling symptoms (NIHSS 0–4/5) until 3 h, and may be a therapeutic option in the same category, in a 3–4.5 h interval. A weaker indication is present (3–4.5 h interval) for patients > 80 years, with severe stroke (NIHSS > 25) and diabetes–stroke history. However, the use in these categories may be effective (29).

Important contraindications are represented by severe hemorrhages/risk of bleeding (e.g., intracerebral hemorrhage; head trauma; coagulopathy; use of anticoagulants or antiaggregant medications; and low platelets count), glycemia <50 or >400 mg/dl, systolic blood pressure > 185 mmHg, or diastolic blood pressure >110 mmHg. Nevertheless, the concomitant administration of anticoagulant or antiaggregant drugs may not be contraindicated if patients take it for solid clinical reasons (29, 96). Besides, its possible interaction with drugs acting on coagulation/aggregation (e.g., direct oral anticoagulants, aspirin, and coumarols), angiotensin-converting enzyme (ACE) inhibitors, may increase the risk of hypersensitivity generated by alteplase (96). Angioedema is a possible, but less common, alteplase side effect. It is probably related to the increase of bradykinin by plasmin activation. Therefore, the coadministration of ACE inhibitors may result in a worsening of macroglossia and angioedema (99). Tenecteplase is a modified alteplase analog. It has a longer half-life, a more specific action on fibrin, and a minor susceptibility to inhibitors (100). In EXTEND-IA TNK trial, tenecteplase (0.25 mg/kg, higher total dose 25 mg) was associated with a better reperfusion, compared to alteplase, in stroke patients until 4.5 h (100). However, tenecteplase (0.4 mg/kg, higher total dose 40 mg) failed to demonstrate superiority with a similar safety profile in a mild stroke prevalent court (101). These results induce guidelines for a lower strength (and maybe temporary) recommendation (IIb) for patients eligible for mechanical thrombectomy and as an alternative in mild stroke (no severe deficits or occlusions). However, in a mild stroke court, Tenecteplase showed to be equal to alteplase (and then it can be used as an alternative). In a tougher clinical context (occlusion of MCA, basilar, carotid), tenecteplase has shown superiority, and these findings will be the object of further studies to maintain or change the indication (29, 100, 101). Tenecteplase has similar adverse events and contraindications compared to alteplase (97). No other tissue plasminogen activators are approved by guidelines (29) (see Tables 4, 5 for details). In patients with an uncertain time of onset, performing a diffusion-weighted MRI (DW-MRI) and fluid-attenuated inversion recovery (FLAIR) sequences is useful: no signal in FLAIR and DW-MRI lesion minor than one-third of middle cerebral artery (MCA) is eligible for fibrinolysis.

**Table 4:** Antiaggregant or fibrinolytic drugs (part I).

	**Bioavailability**	**Half-life**	**Metabolism**	**Protein binding**
**Antiaggregant**				
Aspirin	50% (88)	2–3 h	Hepatic (conjugation)	99%
Clopidogrel	50% minimum	6 h	Hepatic, CYP2C19	98%
Dipyridamole	60%	2.2–15 h	Hepatic (conjugation)	97–99%
**Fibrinolytic**				
Alteplase	-	40 min	Hepatic	-
Tenecteplase	-	90–130 min	Hepatic	-

If not, differently specified information can be found in SmPC.

**Table 5:** Antiaggregant or fibrinolytic drugs (part II).

	**Dosage**	**Elimination**	**Dose adjustment kidney impairment**	**Dose adjustment hepatic disease**
**Antiaggregant**				
Aspirin	Various trial dosages 25–600 mg in monotherapy or combination (*see text)*	Mainly renal	Use with caution. Contraindicated in severe impairment	Use with caution. Contraindicated in severe impairment
Clopidogrel	75 mg	50% urine; 46% feces	Few data available	Few data available
Dipyridamole	200 mg (in combination)	95% feces; 5% urine	No expected pharmacokinetics variations	Use with caution
**Fibrinolytic**				
Alteplase	0.9 mg/kg, maximum dose 90 mg over 60 min	Liver/plasma	Use carefully in hemostatic defects including those secondary to severe hepatic or renal disease	Use carefully in hemostatic defects including those secondary to severe hepatic or renal disease
Tenecteplase	0.25 mg/kg, higher total dose 25 mg	Liver/plasma	Use carefully in hemostatic defects including those secondary to severe hepatic or renal disease	Use carefully in hemostatic defects including those secondary to severe hepatic or renal disease

If not, differently specified information can be found in SmPC.

#### Antiplatelet treatment

##### Acetylsalicylic acid

Acetylsalicylic acid (ASA; also aspirin) exerts its antiplatelet activity by inhibiting cyclooxygenase 1 (COX-1) and depressing thromboxane synthesis (102–104). Aspirin is recommended in stroke patients 24–48 h after onset. In the case of alteplase administration, aspirin is delayed 24 h unless there are other clinical indications or benefits: in this case, it might be useful (29). Hemorrhagic risk should be evaluated carefully, especially in patients who consume multiple medications (29, 104). A systematic review by Sandercock et al. assessed that aspirin 160–300 mg OD (oral) significantly decreased death and complications (except hemorrhage, whose risk was low compared to benefits) (105). Combination therapy of clopidogrel and aspirin shows effectiveness in patients with non-cardioembolic ischemic stroke and NIHSS ≤ 3, not receiving alteplase (beginning in 24 h and continuing for 21 days) (29). The dosage was different in the two main trials. POINT trial randomized patients to a clopidogrel loading dose of 600 mg, followed by clopidogrel 75 mg/day, plus aspirin 50–325 mg/day, for 90 days, compared to aspirin alone. The results showed real effectiveness, but an increase in hemorrhagic risk. There was no benefit in stroke reduction risk after 30 days of treatment, whereas bleeding probability was enhanced after 7 days of treatment (101). A different dose (clopidogrel loading dose 300 mg, and then 75 mg/day, for 90 days, and aspirin 75 mg/day, for the first 21 days) was administered in the CHANCE trial, in comparison with aspirin. This therapeutic scheme generated similar percentages of hemorrhage (0.3%) in the two groups, increasing efficacy. Maybe these results are related to a smaller loading dose or limited duration of dual therapy (106, 107). Furthermore, CYP2C19 Asian polymorphisms may have a role in the development of adverse events, since clopidogrel may be variously transformed in its active metabolite, depending on ultrarapid/slow metabolism (106, 108). In a study by Khatri et al., alteplase was compared to aspirin in people with ischemic stroke, but minor disabling deficits (NIHSS 0-5). Aspirin was superior, despite some study limitations, according to guidelines recommendation. However, the results were not conclusive according to the trial brief duration (109). ASA may be used also in primary (risk factor management) and secondary prevention (50–325 mg daily) (29, 110, 111). ASA is not relevantly metabolized by cytochrome 450 (CYP450) enzymes, and it is not a substrate of transporters. It may compete with other non-steroidal anti-inflammatory drugs (NSAIDs) that also act on COX-1, lowering the ASA effect (112).

Interaction with selective serotonin reuptake inhibitors (SSRI) is also important. These antidepressant drugs reduce the platelet reuptake of serotonin, inhibiting aggregation. Therefore, increased hemorrhagic risk may result from coadministration (104, 112, 113). Another important interaction in this clinical setting involves antihypertensive drugs. In fact, NSAID may suppress renin activity, increase sodium retention, impair the activity of kidney prostaglandins (114), increase the risk of kidney injury, and impair diuretics activity (115). However, other authors suggest that low-dose aspirin does not have an important interaction with kidney and antihypertensive drugs. In fact, COX-2 is principally involved in the production of kidney prostaglandins 2, and aspirin acts on COX-1 (112) (see Tables 4, 5 for details).

##### Clopidogrel

Clopidogrel is an antiplatelet agent, which acts as a prodrug. The active compound is generated by CYP450 metabolism, and then, it blocks P2Y_12_ platelet receptor. Through this pharmacodynamic action, it prevents the binding of adenosine diphosphate (ADP) to the same target (116, 117). Activation of P2Y_12_ in physiological conditions leads to the activation of a pathway that determines the release of granules, a stronger platelet aggregation, and the activation of the glycoprotein IIb/IIIa receptor (GP IIb/IIIa) (118). Clopidogrel (75 mg) may be used in non-cardioembolic stroke or transient ischemic stroke (TIA) as secondary prevention for patients with risk factors. Antiplatelet treatment is strongly recommended by guidelines (10, 29). In this setting, aspirin (50–325 mg) alone, clopidogrel (75 mg) alone, and aspirin (25 mg) + dipyridamole (200 mg) are the main options. In non-cardioembolic stroke patients with NIHSS ≤ 3 or high-risk TIA (ABCD^2^ score ≥4), double antiplatelet therapy (DAPT) with aspirin plus clopidogrel in 12–24 h from clinical insurgence is the right option (if alteplase has not been administered). This therapy should be maintained up to 21–90 days, then switching to single antiplatelet therapy: a longer treatment period would result in increased bleeding risk without benefit (29, 111). Clopidogrel is mainly transformed by CYP2C19 that produces its active metabolite (R-130964), a thiol derivative. CYP1A2, 2B6, and 3A4 have a certain role in this process (116). Therefore, inhibition (e.g., some proton pump inhibitors [PPI], some SSRI, and some antifungal medication resulting in reduction of active metabolite production) or induction (rifampicin) of CYP2C19 may alter therapeutic action (116, 118). Bleeding or side effects related to reduced platelet action are the most common side effects (116, 119) (see Tables 4, 5 for details).

##### Antiplatelet combination therapy and other options

The ASA was tested in clinical trials in coadministration with dipyridamole. Dipyridamole has both, vasodilator and antiplatelet effects. This drug inhibits adenosine reuptake from red blood cell precursors and inhibits cyclic-3'5'-adenosine monophosphate (cAMP) phosphodiesterase. Therefore, cAMP accumulates and exerts an antiaggregant activity. Vasodilator effect is generated by cyclic-3'5'-guanosine monophosphate (cGMP) phosphodiesterase inhibition by dipyridamole. It results in an increase of cGMP and of its action on blood vessels (120). Some trials observed a better efficacy of aspirin plus dipyridamole compared to ASA alone in stroke secondary prevention (121). Clopidogrel plus ASA showed a better efficacy compared to ASA plus dipyridamole as antiaggregant therapy (122). However, ASA + clopidogrel DAPT has very specific indications, and dipyridamole + ASA is considered a good therapeutic option (111). Triple therapy (ASA + clopidogrel + dipyridamole) has been revealed to be dangerous, without any benefit, and it is contraindicated by guidelines (29, 122). An important statement is that, in case of cardiac disease/embolic origin of stroke, anticoagulants (direct anticoagulants or warfarin, varying in different indications) have a major role in secondary prevention (especially in atrial fibrillation). Nevertheless, antiplatelet agents may be used alone or in combination with anticoagulants, depending on etiology (111). Glycoprotein IIb/IIIa inhibitors are not useful in this pathology. Tirofiban and eptifibatide efficacy have not been observed, and abciximab administration may even be dangerous, especially when associated with alteplase (29). A systematic review by Ciccone et al. showed an increase of hemorrhagic risk without benefit in terms of clinical effectiveness. However, the majority of the studies included regarded abciximab. Although its dosage and specific indications have not been described, tirofiban showed an interesting potential in stroke (123, 124). Nevertheless, other authors denied eptifibatide and tirofiban safety and effectiveness. Guidelines used a IIb recommendation on tirofiban and eptifibatide, assessing the need of further trials and analysis (29, 125). Ticagrelor, a P2Y_12_ antagonist, has an uncertain role in stroke secondary prevention. Guidelines talk about a IIb recommendation, according to THALES trial. In fact, ticagrelor plus aspirin showed a better outcome of death stroke, compared to aspirin alone. The study included patients with a mild to moderate pathology assessment (NIHSS ≤ 5) or TIA. The risk of hemorrhage was increased with ticagrelor (111, 126). Ticagrelor alone was inferior to aspirin in SOCRATES trial in the management of minor acute stroke, with comparable safety outcomes (127) (see Tables 4, 5 for details).

## Discussion

The AVS represents a dramatic clinical situation causing an important feeling of fear in the patients who experience this event. A rapid and correct diagnosis must be the main goal when evaluating a patient with AVS. In fact, a wrong or delayed diagnosis does not allow to formulate an effective treatment plan and may cause devastating consequences for the patient's health. The initial phase of AVS is mainly managed by general practitioner and emergency room doctors. Patient's complained symptoms show a wide variability, including vertigo, vomiting/nausea, dizziness, headache, confusion, hearing alteration, and neurological deficits. This makes difficult to achieve a correct diagnosis with a basic clinical evaluation, making it necessary to perform specialistic instrumental evaluation and/or imaging investigation. As mentioned above, CT and MRI represent two fundamental radiological aids that allow to detect the eventual signs of ischemia or hemorrhage. According to recent studies, VN occurs more frequently in people over the age of 70 years (128, 129). Its exact etiology still remains unclear. Regarding viral infection of the vestibular nerve, it is considered that viruses causing infections of the upper respiratory tract, such as influenza virus, adenovirus, HSV, cytomegalovirus, Epstein-Barr virus, parainfluenza virus, and, recently, SARS-CoV-2, could be VN related, because associations with preceding or concurrent viral infection in the upper respiratory tract occur in 43% to 46% (130). Among them, HSV type 1 is the most common cause of viral infection of the vestibular nerve and ganglion. Recently, *in vivo* work demonstrated that HSV infection can induce VN and sudden deafness in a mouse model (131). Immunological mechanisms have also been suggested as possible causes of VN. A pathological CD4/CD8 quotient, which appears in 48% of NV cases, further supports a causal immunological origin (11). The characteristic clinical features of VN are abrupt true-whirling vertigo, lasting for more than 24 h, with nausea and vomiting, in middle age without cochlear symptoms and other neurological symptoms and signs. Prodromal dizziness lasting few minutes, in the few days just before the full onset of symptoms, may precede prolonged spontaneous vertigo in about 25% of patients (132). Unlike BPPV and MD, the clinical features of VN can make this pathological entity resemble PCS. In fact, during BPPV the vertigo is caused by head movement, and exacerbations of MD typically present specific audiological symptoms associated with vertigo (133–135). For these reasons, VN must be considered as the main pathological condition that may mimic a CNS ischemic stroke. Various treatments have been reported for VN, which can be largely divided into symptomatic therapy, specific drug therapy, and vestibular rehabilitation. Vestibular suppressants are widely used because they are effective against dizziness, nausea, and vomiting. During the acute stage of VN, an intramuscular or intravenous route for vestibular suppressants and antiemetics is usually preferable because of severe nausea and decreased gastric motility. However, most vestibular suppressants can have sedative effects, so they should not be used when patients are engaged in activities that require a high level of alertness, such as driving, operating machinery, or participating in athletic activities. Regarding specific drug therapy, steroid therapy has been reported to relieve dizziness and promote VC in VN. Methylprednisolone is much more effective than placebo in reducing vertiginous symptoms in patients with acute vestibular vertigo, and early treatment of acute VN with high doses of glucocorticoids accelerates and improves the recovery of vestibular function (136). Nevertheless, a recent meta-analysis by Leong et al. concluded that corticosteroids appear to have short-term benefits in canal paresis but no long-term benefits in canal paresis and symptomatic recovery (137). Concerning VN long-term treatment, the gold standard for therapy is represented by vestibular rehabilitation. Its targets are to improve vertigo, gaze stability, postural stability, and daily living activities through VC and central neuroplasticity. Vestibular rehabilitation consists of a dynamic compensation of vestibular reflexes that are activated by movement, and it is composed of adaptation, habituation, and substitution. Vestibular rehabilitation exercises are safe, highly therapeutic, highly cost-effective, and significantly hasten vestibulospinal compensation in patients with VN (138–140). Balance and gait exercises significantly reduce the time required for vestibulospinal compensation. Voluntary eye movements, active head movements, goal-directed movements, and walking should be encouraged to restore postural control and balance as soon as possible. Patients with VN should exercise for at least 30 min, 3 times a day (132). An interesting therapeutic opportunity is also offered by nutraceuticals, especially in the intercritical phases of the disease or in the recovery of residual imbalance in some subjects. These are safe and effective compounds that can be administered without associated drugs or in combination to decrease their dosage (12, 141). The prognosis in patients with VN is generally good, but residual dizziness may remain in some patients after the acute phase, similar to persistent disabling imbalance after successful repositioning maneuvers for BPPV. This can be due to many factors, including inadequate central compensation, incomplete peripheral recovery, and psychophysiological and psychological characteristics. The decreasing postural control can affect the quality of life, contributing to falls and psychological problems (142). In contrast, approximately 20% of ischemic events involve tissue supplied by the posterior circulation territory, such as the cerebellum and brainstem. The incidence of cerebellar infarction in larger series of patients with stroke is approximately 1.5 %, with an average patient age of about 60 years (143). Tissue plasminogen activators and antiplatelet treatment represent the two principal categories of drugs for the prompt treatment of cerebral ischemic stroke. Regarding AVS, the main clinical goal is to obtain a fast and correct differential diagnosis between VN and cerebellar stroke. Indeed, the best therapeutic effects can be greatly reduced when stroke treatment is administered late. For this reason, when evaluating a patient with AVS, the crucial question is to clarify the correct etiology of the symptoms. Dizziness/vertigo is a common symptom in patients with isolated strokes of the cerebellum, usually with other neurological symptoms and signs. However, the diagnosis of isolated vertigo from brainstem and cerebellar stroke has increased markedly with recent developments in clinical neurotology and neuroimaging. Patients with infarction in the AICA territory may have isolated recurrent vertigo, acute hearing loss, and/or tinnitus as the initial symptoms (144). This particular clinical entity is also well defined as “labyrinthine infarction.” The acute hearing loss is usually caused by the thrombotic narrowing of the AICA or the basilar artery at the orifice of the AICA. Through this mechanism, decreased blood flow in the affected AICA might cause either a transient episode of selective ischemia to the inner ear, resulting in isolated prodromal vertigo, or permanent damage to the widespread areas involving the middle cerebellar peduncle, lateral pons, and anterior cerebellum, resulting in acute hearing loss and prolonged vertigo in addition to other central symptoms and signs (145). The apical region of the cochlea is particularly vulnerable to vascular damage and, therefore, low-frequency hearing loss is common in inner ear ischemia (146). To date, at least eight subgroups of AICA infarction have been identified, according to the pattern of neurotological presentations, among which the most common pattern of audiovestibular dysfunction is the combined loss of auditory and vestibular functions (147). Ischemia of the PICA usually produces no auditory symptoms, because it does not perfuse the auditory tract, generally. However, PICA infarction may rarely be associated with acute hearing loss as the internal auditory artery sometimes originates from the PICA or directly from the basilar artery (148). For a proper management, in all cases of AVS, it is very important to know when a patient needs an urgent brain scan, and what role does neuroimaging play in diagnosis. Because central signs, such as spontaneous vertical ny, direction-changing gaze-evoked ny, perverted head shaking ny, or severe postural instability with falling, are known to have high specificity, but low sensitivity, for detecting a central cause of vertigo, isolated acute vertigo due to cerebellar infarction at the bedside remains a diagnostic challenge. The cerebellum plays an important role in maintaining body posture, regulating the muscle tension associated with postural movements, and coordinating voluntary movements (149). The vermis is involved in the coordination of eye and body movements, provides visual and auditory input related to balance, and is involved in vestibular system regulation and in maintaining the position of the head (150). Unfortunately, pharmacotherapy and conventional rehabilitation treatments, including core strength exercises, visual feedback training, neurodevelopmental therapy, and proprioceptive neuromuscular facilitation, performed unsatisfactory results on balance recovery among stroke patients (151).

## Conclusion

The therapeutic approach to AVS conditioned by the ability to make a correct differential diagnosis and as certain as possible from an etiological point of view. While we have sufficient tools to identify the location and mechanism of the damage, it is not always possible to have immediate evidence of the etiology. This can affect the accuracy of the therapeutic choice by forcing less specific therapies from a causal point of view. Furthermore, the correct pharmacological action lays the foundations for obtaining an effective VC. At the same time, another determining element, directly linked to the therapeutic choice, is the time factor. In fact, the precocity of intervention can guarantee, in general, better outcomes and, in some specific cases, can avoid the evolution toward much more critical clinical pictures and, in a significant percentage, toward non-compensation or transformations into persistent dizziness (152).

## Author contributions

PV, FG, and GM: conceptualization, methodology, investigation, data analysis, visualization, and writing—original draft. AA, DP, and AS: investigation and data analysis. AC, EB, and VR: investigation, data analysis, and project administration. MR and GC: investigation, visualization, and software. All authors contributed to the article and approved the submitted version.

## Conflict of interest

The authors declare that the research was conducted in the absence of any commercial or financial relationships that could be construed as a potential conflict of interest.

## Publisher's note

All claims expressed in this article are solely those of the authors and do not necessarily represent those of their affiliated organizations, or those of the publisher, the editors and the reviewers. Any product that may be evaluated in this article, or claim that may be made by its manufacturer, is not guaranteed or endorsed by the publisher.
